# Predicting the spatial abundance of *Ixodes ricinus* ticks in southern Scandinavia using environmental and climatic data

**DOI:** 10.1038/s41598-019-54496-1

**Published:** 2019-12-02

**Authors:** Lene Jung Kjær, Arnulf Soleng, Kristin Skarsfjord Edgar, Heidi Elisabeth H. Lindstedt, Katrine Mørk Paulsen, Åshild Kristine Andreassen, Lars Korslund, Vivian Kjelland, Audun Slettan, Snorre Stuen, Petter Kjellander, Madeleine Christensson, Malin Teräväinen, Andreas Baum, Kirstine Klitgaard, René Bødker

**Affiliations:** 1 Department of Veterinary and Animal Sciences, Faculty of Health and Medical Sciences, University of Copenhagen, Frederiksberg, Denmark; 2 Department for Diagnostics and Scientific Advice, National Veterinary Institute, Technical University of Denmark, Lyngby, Denmark; 3 Department of Pest Control, Norwegian Institute of Public Health, Oslo, Norway; 4 Department of Virology, Norwegian Institute of Public Health, Oslo, Norway; 5 Department of Production Animal Clinical Sciences, Norwegian University of Life Sciences, Oslo, Norway; 6 Department of Natural Sciences, University of Agder, Kristiansand, Norway; 7 Sørlandet Hospital Health Enterprise, Research Unit, Kristiansand, Norway; 8 Department of Production Animal Clinical Sciences, Section of Small Ruminant Research, Norwegian University of Life Sciences, Sandnes, Norway; 9 Department of Ecology, Wildlife Ecology Unit, Swedish University of Agricultural Sciences, Grimsö, Sweden; 10 Department of Applied Mathematics and Computer Science, Technical University of Denmark, Lyngby, Denmark

**Keywords:** Ecological epidemiology, Infectious diseases, Machine learning

## Abstract

Recently, focus on tick-borne diseases has increased as ticks and their pathogens have become widespread and represent a health problem in Europe. Understanding the epidemiology of tick-borne infections requires the ability to predict and map tick abundance. We measured *Ixodes ricinus* abundance at 159 sites in southern Scandinavia from August-September, 2016. We used field data and environmental variables to develop predictive abundance models using machine learning algorithms, and also tested these models on 2017 data. Larva and nymph abundance models had relatively high predictive power (normalized RMSE from 0.65–0.69, R^2^ from 0.52–0.58) whereas adult tick models performed poorly (normalized RMSE from 0.94–0.96, R^2^ from 0.04–0.10). Testing the models on 2017 data produced good results with normalized RMSE values from 0.59–1.13 and R^2^ from 0.18–0.69. The resulting 2016 maps corresponded well with known tick abundance and distribution in Scandinavia. The models were highly influenced by temperature and vegetation, indicating that climate may be an important driver of *I. ricinus* distribution and abundance in Scandinavia. Despite varying results, the models predicted abundance in 2017 with high accuracy. The models are a first step towards environmentally driven tick abundance models that can assist in determining risk areas and interpreting human incidence data.

## Introduction

Within the last decades a rise in tick-borne diseases in Europe such as tick-borne encephalitis (TBE), and Lyme borreliosis (LB)^1–3^ has caused concerns that climate change may be altering the dynamics of vectors and their associated diseases^4,5^. In Europe, and especially in Scandinavia, the hard tick *Ixodes ricinus* is the most common vector of tick-borne pathogens^2,6,7^. Scandinavia constitute the northern-most range of *I. ricinus* in Europe^7,8^, and LB and TBE have been increasing in both Norway and Sweden^8–12^, whereas in Denmark, LB has long been endemic^2^, but TBE has only been found in two geographical areas^13,14^.

Although human behavior may affect the risk of tick exposure, tick abundance and pathogen prevalence also determine human tick-borne disease incidence^15^. Many studies have shown how tick abundance not only influences human exposure to ticks but also has an impact on the relative prevalence of pathogens within the ticks^16–19^. In Sweden, Jaenson *et al*.^16^ found a significant correlation between *I. ricinus* nymph density and prevalence in ticks of *B. burgdorferi* sensu lato (group of spirocheates causing LB), indicating that nymph density could be used to assess risk of human exposure to *B. burgdorferi* s.l. Mysterud *et al*.^20^ found that human LB incidence in Norway was higher in areas with high *I. ricinus* abundance, and Jensen *et al*.^21^ found a correlation between neuroborreliosis incidence and tick densities in Denmark.

In addition to climate, abundance and distribution of *I. ricinus* may also be affected by factors such as land cover, landscape composition, and availability of host species^1,3^. Several studies have investigated tick distribution and abundance in Scandinavia. Although conclusions differ regarding a latitudinal range expansion for *I. ricinus* in Norway^6,8,22^, Jaenson *et al*.^7^ found *I. ricinus* to have expanded their northern range in Sweden and found an increase in tick abundance over time within their well-established range. High abundances in Norway are found along the coastline in the southeast to approximately 65.3°N^6,8,9,11,12,22^, whereas high abundance are found in south and central Sweden^7^. In Denmark, *I. ricinus* has been widespread for years, but an increase in abundance has been reported throughout the country since the late 1980’s^23^. This rise in abundance in Scandinavia has been ascribed to both climate change and the resulting effects on vegetation but also an increase in the number of the main host for adult tick species in Scandinavia – the roe deer (*Capreolus capreolus*)^3,7,23^.

*I. ricinus* has been found to have higher abundance in forest habitats^24–26^, as these habitats provide environmental and climatic conditions optimal for tick survival^24,26,27^. The forest configuration also affect tick abundance, as forest fragmentation and the resulting increasing edge zone provide cover and forage for several tick host species, thus elevating local abundances of these species^26,28^.

Although host density is important when determining drivers of tick abundance, data on host species can be hard to obtain, especially for larger regions. Many studies have, however, been able to link environmental and climatic variables to tick presence and abundance^16,21,29–34^. Finding and validating links between tick abundance/distribution and environmental and climatic variables, may enable us to develop models that can predict to un-sampled regions and potentially also predict future scenarios of abundance and distribution. Several models have used environmental variables to predict emergence of TBE^35^ and TBE exposure^36^ as well as the potential future distribution of *I. ricinus* in Europe, North Africa and the Middle East^37–39^.

Modelling vector abundance and distribution may aid the responsible authorities in targeting areas at risk for disease outbreaks or increases in incidence of already established diseases. We have previously modelled the geographical distribution of *I. ricinus* nymphs in southern Scandinavia, using presence/absence data from the largest uniform data set from Scandinavia to date, environmental variables, and machine learning (ML) techniques^34^. Here we apply abundance data from the same nymph data set and similar additional datasets for larvae and adults and ML techniques to model abundance of *I. ricinus* in Scandinavia. Whereas spatial tick models can predict the geographical range of ticks and general habitat occurrence, abundance models provide a more detailed measure of habitat suitability and a measure of high exposure zones, as well as providing data for classical epidemiological modelling (such as R_0_ models). We wanted to create tick abundance models with high predictive power using environmental variables. Our main focus was the predictive ability of the models, not identifying risk factors or quantifying the effect of the different variables on tick abundance. We had 2 distinct aims with this study. First, we wanted to create spatial maps of tick abundance in Scandinavia for the year 2016. Secondly, we wanted to test if these models could also be used to predict tick abundance for the same time period the following year, which would show that our modelling concept could be used to predict tick abundance for the same time period in other years as well. These maps can then be used with human and animal health data to assess the spatial risk of tick bites and thus the risk of getting infected with a tick-borne pathogen. We here present the first maps of *I. ricinus* abundance in southern Scandinavia for a specific period in a specific year, created using ML techniques and environmental variables.

## Methods

### Stratification of study region and site selection

**Figure 1:**
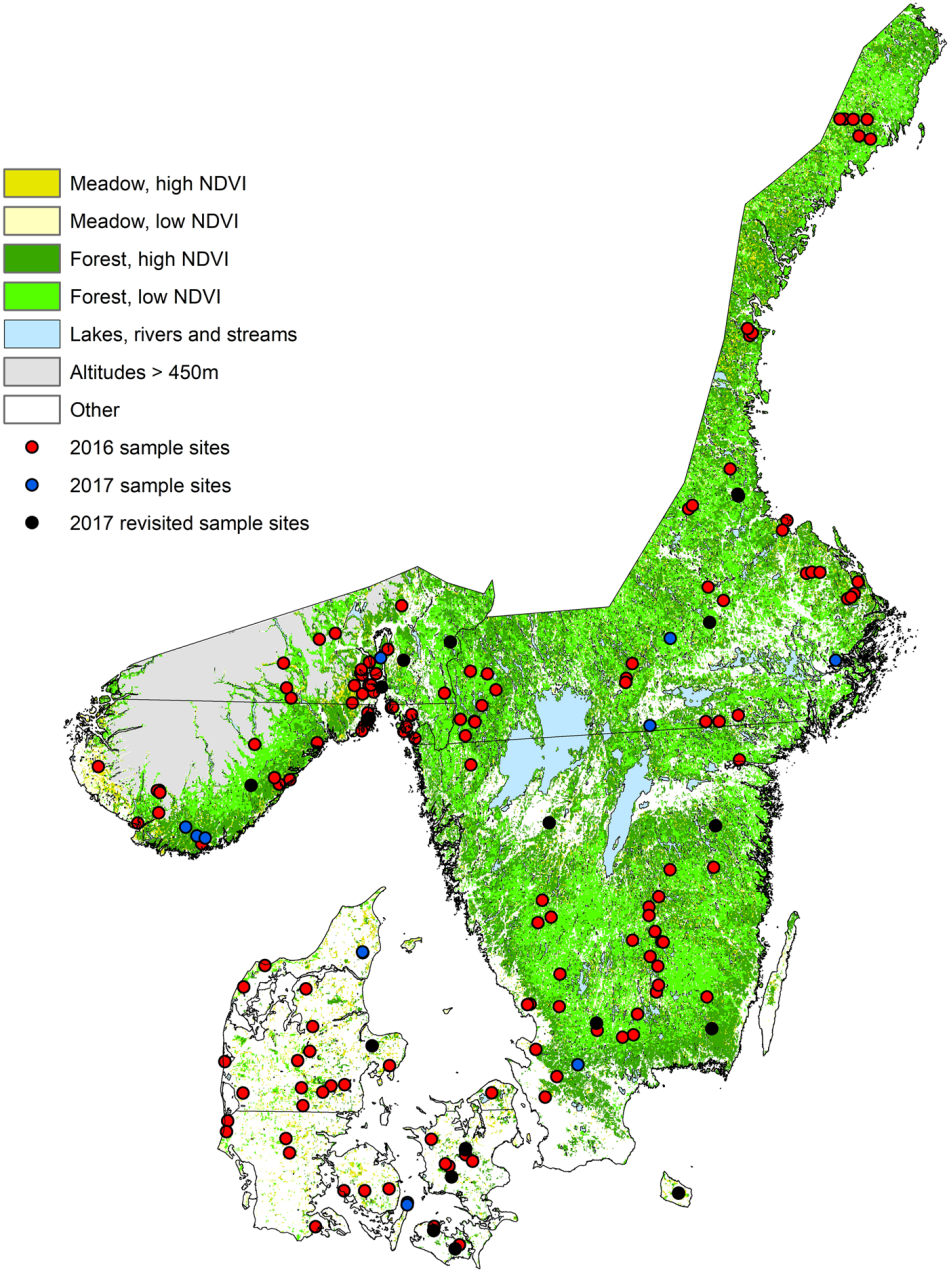
The stratification of the study area in southern Scandinavia. The map depicts the 159 sample sites from 2016 (red) and the 12 new sample sites from 2017 (blue), with the revisited sites from 2016 (black) depicted. Habitat definitions are described in Table 1. The lines through each country divide the country into equally sized north and south strata. Only parts of Norway and Sweden were included in the field study, due to logistics. NDVI is the normalized difference vegetation index.

The stratification of the study region has been described in details in Kjær *et al*.^34^. Our study region included all of Denmark, southern Norway and south-eastern Sweden (Fig. 1). We excluded all altitudes above 450 m, where we expected very low abundance/absence of ticks. We used Fourier processed satellite imagery of the maximum normalized difference vegetation index (NDVI^40^) and land cover data from Corine (all 1 × 1 km resolution^41^). After dividing each country within the study region into equally sized north and south parts, we divided them further into “high” NDVI (>median value) and “low” NDVI (≤median value). We then divided these stratified regions into forest and meadow, using the Corine Land Cover classes defined in Table 1. No other land cover classes, e.g. urban and agricultural areas, were sampled for ticks (or predicted by the resulting model, Fig. 1).

**Table 1:** Stratification of land cover types for selecting field sites to survey *I. ricinus* within southern Scandinavia.

Stratification definition	Corine land cover definition
Forest	Broad-leaved forest
	Coniferous forest
	Mixed forest
Meadow	Land principally occupied by agriculture with significant areas of natural vegetation
	Natural grasslands^*^
	Moors and heathland
	Transitional woodland-shrub

^*^This land cover type was not found at any of the study sites.

We used R 3.4.2^42^ (sampleStratified in the raster package) to randomly select 30 first priority sample sites consisting of 80% forest and 20% meadow in each country^34^. For each first priority site, we randomly selected 10 alternative sites (if access to first priority sites proved impossible or if not enough tick nymphs could be collected, see below) ordered in priority after smallest distance to original site. For meadows, we additionally created 10 alternative forest-sites, if it proved impossible to collect ticks in meadows.

We additionally chose 20 random sites along the Oslo Fjord in Norway (with 10 alternatives), as we were interested in investigating tick abundance along the Oslo Fjord in greater detail.

### Field study

We measured tick abundance between 15. August and 30. September, 2016, within the hours of 11–16, using a 100 m north and a 100 m east facing transect at each site^34^. In both directions along each transect, we “dragged”^43^ a white flannel cloth (1.05 × 1.15 m, containing lead weights in one end), removing and counting tick instars every 50 meters. This study was a part of a larger study to collect ≥600 nymphs from 30 sites in each country for pathogen detection. As some sites had no or low nymph abundance, an alternative site was chosen for nymph collection while keeping the abundance data from the original site, resulting in more than 30 sites with abundance measures per country (Fig. 1). In 2017, using the exact same procedure as in 2016, we measured abundance at 10 sites in each of the three countries between 15. August to 30. September. Six of those 10 sites were previously visited in 2016; the three sites with the lowest (excluding zeros) and the three sites with the highest tick nymph abundance, respectively (Fig. 1). We did this to gauge whether the relative differences in abundances would be consistent or simply a result of annual variation, and we chose nymphs to be the measure for new site selection as they were not as patchily distributed as larvae (adult counts were too low and not used as a measure). We used Spearman’s rank correlation to compare the number of instars between the two years.

The remaining four of the ten sites were completely new sites in each of the three countries, and selection of these sites were based on areas of interest (Fig. 1). We planned to use all the 2017 sites to test the boosted regression tree (BRT) abundance models (based solely on 2016 data), but kept the validation of the revisited and new sites separate, as the 18 revisited sites were part of the randomized site selection scheme while the 12 new sites were not randomly selected.

### Abundance modelling

We used R 3.4.2 (packages caret and gbm) to run four BRT models on the log-transformed abundance data (total count of all 400 m transects for each site) from 2016 for larvae, nymphs, adult males and adult females respectively, using 92 environmental predictors (rasters with a pixels size of 1 km^2^, Table 2). We created separate models for each instar as pathogen prevalence varies greatly between tick instars and thus each instar poses a different disease risk to humans and animals^44,45^. BRT is a ML technique using regression trees and gradient boosting^46^, that produces predictions of a response variable, in our case log(abundance). The resulting models can then be used to predict tick abundance in un-sampled areas if the predictor variables are available for these areas.

**Table 2:** The environmental predictors used in the boosted regression tree models to predict tick abundance in the modelled Scandinavian region.

Source	Variables
Modis (Fourier transformed), 2001–2012^* ^^40^	Middle infra-red
	Daytime land surface temperature
	Night-time land surface temperature
	NDVI
	EVI
WorldClim 1.4, 1960–1990^61^	Altitude
BioClim (WorldClim), 1960–1990^62^	BIO1: Annual mean temperature
	BIO2: Mean diurnal range (mean of monthly (max temp – min temp))
	BIO3: Isothermality (BIO2/BIO7)*100
	BIO4: Temperature seasonality (standard deviation *100)
	BIO5: Max temperature of warmest month
	BIO6: Min temperature of coldest month
	BIO7: Temperature annual range (BIO5-BIO6)
	BIO8: Mean temperature of wettest quarter
	BIO9: Mean temperature of driest quarter
	BIO10: Mean temperature of warmest quarter
	BIO11: Mean temperature of coldest quarter
	BIO12: Annual precipitation
	BIO13: Precipitation of wettest month
	BIO14: Precipitation of driest month
	BIO15: Precipitation seasonality (coefficient of variation)
	BIO16: Precipitation of wettest quarter
	BIO17: Precipitation of driest quarter
	BIO18: Precipitation of warmest quarter
	BIO19: Precipitation of coldest quarter
Harmonized World Soil Database v 1.2 (FOA, IIASA), 2009^63^	Soil types, depicted by Soil Mapping Unit Code of major soil group (FAO-90 soil classification system)
Corine Land Cover (2006) raster data^41^ Corine Land Cover (2006) raster data (100 m^2^)^41^	European inventory of land cover in 44 classes European inventory of land cover in 44 classes

^*^For each of the variables, the Fourier processing output includes mean, minimum, maximum, variance in raw data, combined variance in annual, bi-annual, and tri-annual cycles as well as amplitude, phase and variance of annual, bi-annual and tri-annual cycle.

All predictors come as raster files with a resolution of 1km^2^, except the 100 × 100 m Corine Land Cover raster. NDVI is the normalized difference vegetation index; EVI is the enhanced vegetation index.

We used packages raster and gdal to calculate and add 12 more predictor variables. As a measure of habitat fragmentation, we used the 1 km^2^ Corine Land Cover raster to calculate the percentage of the surrounding eight pixels identical to the land cover class, to assess if a particular land cover pixel was part of a large patch (low fragmentation) or if it was a solitary patch (high fragmentation). We only calculated the surrounding cover percentages for land cover types in which tick abundance was measured, however all cover types surrounding a pixel were used for the calculation, resulting in six additional predictor variables. To assess the effect of habitat edges, we used a higher resolution Corine Land Cover raster (100 m^2^) to calculate the amount of edge of the above cover types (in 100 m’s) within a 1 km^2^ pixel. We looked at each 100 m^2^ pixel and assessed the eight surrounding pixels. If any of these eight pixels were of a different cover type than the pixel being investigated, the latter pixel would be considered an “edge” pixel. We summed up the values to correspond to a 1 km^2^ resolution, resulting in six 1 km^2^ rasters for each land cover type.

We used a tuning grid to optimize model parameters, varying interaction depth, learning rate and minimum observations per node^46^, whereas number of trees was kept constant at 1,500 trees. We performed a stratified 5-fold cross-validation (CV) with 10 repetitions to validate our models, to select the optimal tuning parameter values and to estimate the prediction error, i.e. the normalized root-mean-square error (NRMSE, RMSE divided by the standard deviation of the observed data). A 5-fold CV, randomly divides the data into 5 equally-sized folds, and fits a model excluding one fold at a time, using this excluded fold as an evaluation test set. The 10 repetitions, resulted in ca. 1550 predictions for each model (159/5 × 5 × 10). Using a 5-fold CV process, ensures that the model is continuously built on a subset of the data and evaluated by applying it to the remaining “unseen” data. The aim of the CV process is to tune model parameters and to evaluate how well the model can predict unseen data. Once a final model with optimal tuning parameters for each instar was selected based on 2016 data and CV results, we used these final models based on the entire dataset to predict instar abundance in the entire study area for 2016. We used ArcMap 10.6.1^47^ to depict the resulting abundance maps.

To test how well our models could predict abundance at sites in 2017, we first used the final 2016 instar models to predict abundance at the new 2017 sites, and evaluated model fit by comparing the modelled abundance to the observed abundance using NRMSE and R^2^. To test predictive power for the revisited sites in 2017, we reran the 2016 models for larvae and nymphs (complete with a 5-fold CV), but additionally used a leave-one-out (LOO) approach by running each of the instar models 18 times, each time leaving out a single 2016 site that was revisited in 2017. We then used each of these 18 new 2016 models to predict instar abundance at the 2017 site that was left out in the particular 2016 model. We evaluated model fit by calculating NRMSE and R^2^ of the observed and predicted abundance at the 2017 sites. We used the LOO approach since leaving these 2016 data in the model to predict abundance at the revisited sites in 2017 would bias the predictions, as these sites would already be known to the model. We used the LOO approach instead of removing all 18 points at once, since this would reduce our sample sizes and could impair the ML approach.

### Simple spatial interpolation

We additionally wanted to compare the ML models produced to simple spatial interpolation methods to test, whether ML models actually provided more information. We used Inverse Distance Weighting interpolation (IDW)^48^ in R 3.4.2 (packages gstat and spm), which predict values to unmeasured locations based on the values of the surrounding measured locations (the search neighborhood). The IDW method is purely spatial and assumes that the influence (weight) of the measured points diminishes with distance away from the unmeasured point, and that the weight of these points are proportional to the inverse of the distance from the unmeasured point raised to a power^48^. We used a tuning grid to optimize the IDW interpolations, by varying the power as well as the size of the search neighborhood. We furthermore performed a 5-fold CV with 10 repetitions to validate the resulting interpolations and to compare them to our BRT models. We depicted the resulting interpolation maps in ArcMap 10.6.1^47^.

## Results

### Field study

**Figure 2:**
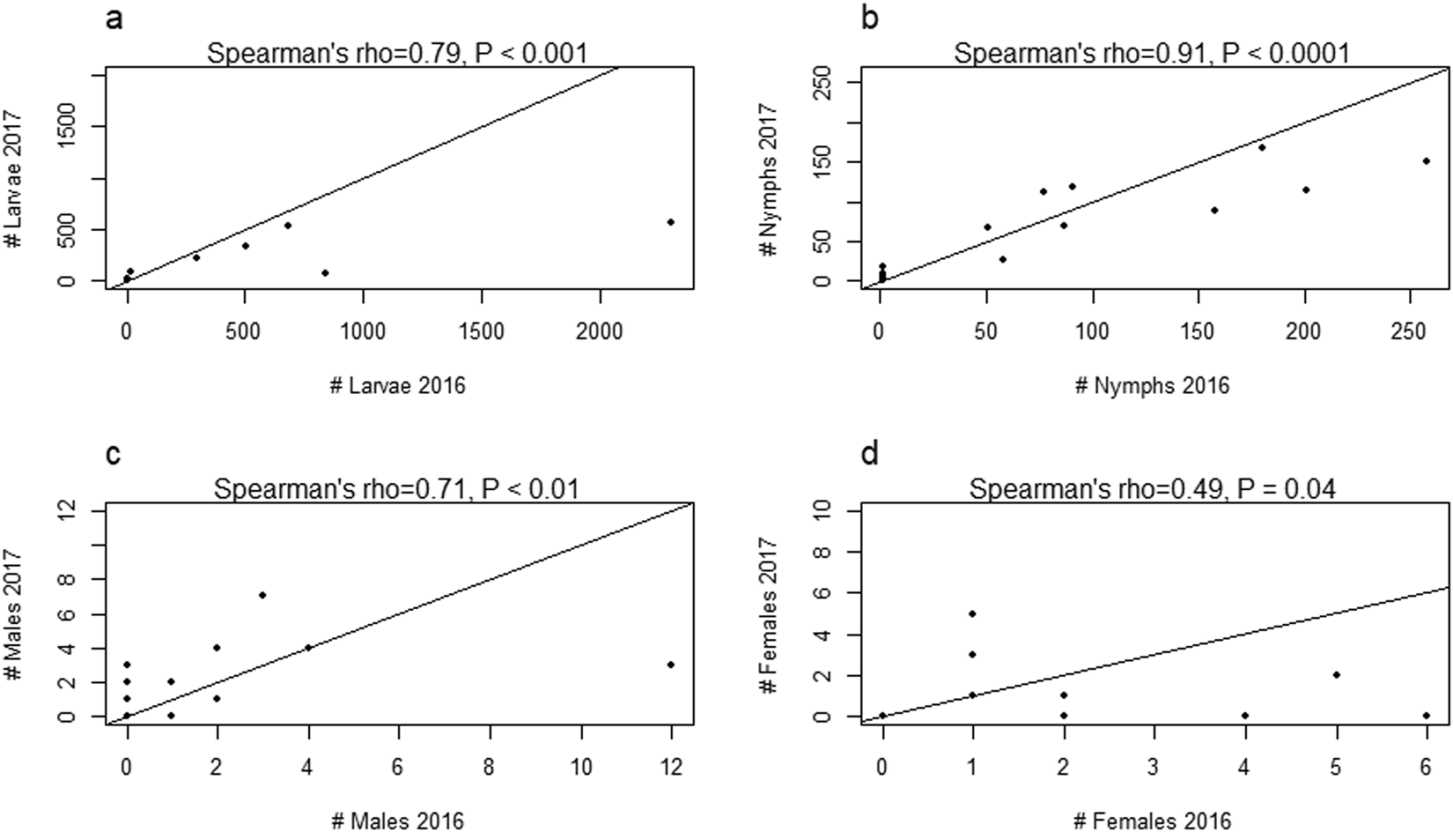
Comparison of the observed *I. ricinus* abundances from 2016 and 2017. Observed summed *I. ricinus* abundances for the year 2016 plotted against the summed abundances for the year 2017 for the 18 sites that were revisited in 2017. (**a**) Larvae, (**b**) nymphs, (**c**) adult males, (**d**) adult females. The black line depicts a 1:1 relationship between counts from 2016 and 2017. Spearman’s rho above each graph depicts the results from the Spearman Rank correlation. Not all points are visible as some counts had the same value in 2017 as in 2016 (for example, 8 sites had zero counts of adult males and females for both 2016 and 2017; this number was 4 and 3 for larvae and nymphs, respectively).

In 2016, we measured abundance at 37, 47 and 75 sites in Denmark, Norway and Sweden respectively (Fig. 1). In Denmark, 70% of these sites were in forested habitats, with 30% of the sites in meadow habitats. In Norway, 80% of the sites were in forested habitats and 20% of the sites were in meadow habitats, whereas in Sweden, 79% of the sites were in forested habitats and 21% were in meadow habitats. The 159 sites present 63.6 km of dragged transects^34^. By revisiting 6 sites in each country in 2017, we found that despite variation between the years in the absolute number of instars, the numbers correlated well between years, where sites with low and high abundance in 2016 also had low and high abundance in 2017 (Spearman’s rho ranging from 0.49–0.91 and all P < 0.05, Fig. 2). Larvae and nymphs had higher abundance in 2016 than in 2017, whereas a pattern was not clear for adult ticks (Fig. 2).

### Abundance modelling

**Figure 3:**
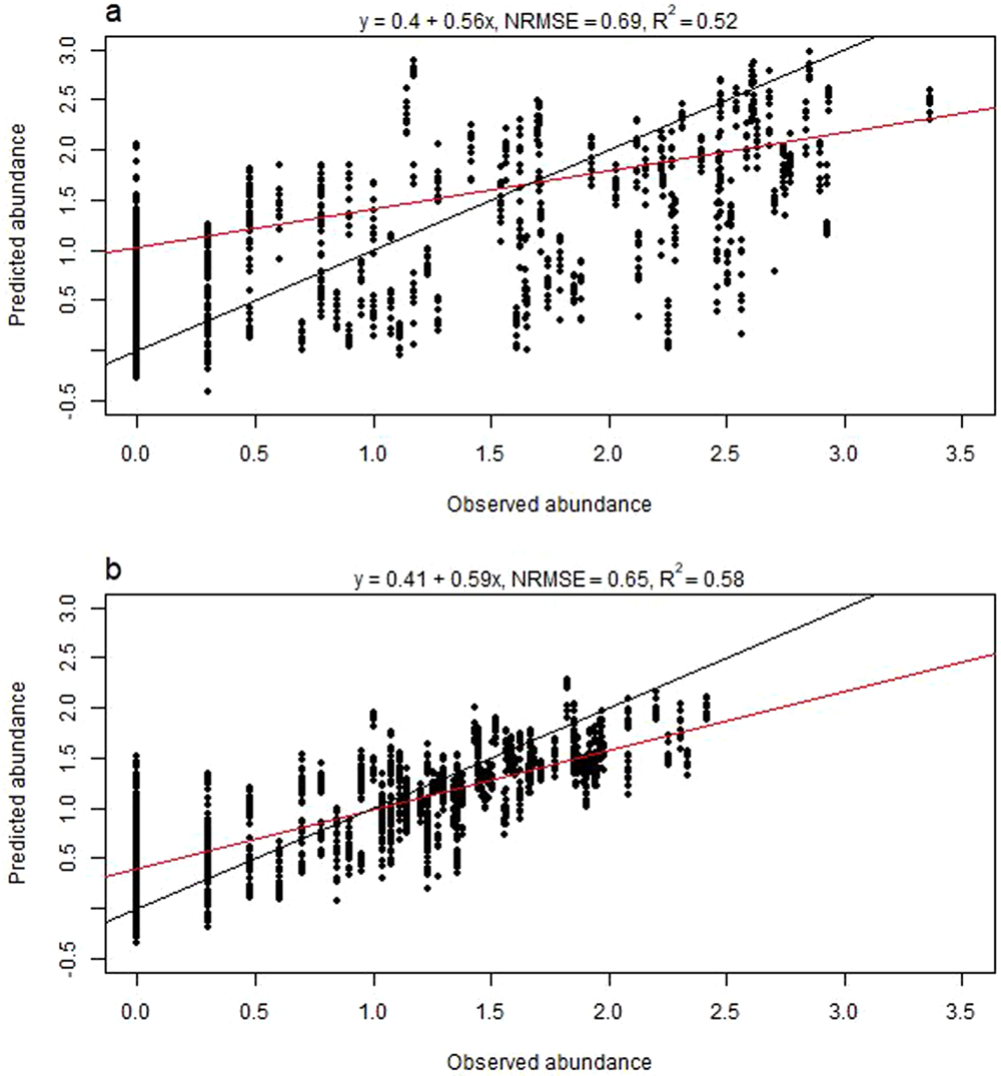
Observed versus predicted abundances of *I. ricinus* larvae and nymphs. Observed abundances (log(N + 1)) plotted against predicted abundances (5 folds, 10 repeats, ca. 1550 predictions) for (**a**) *I. ricinus* larvae and (**b**) *I. ricinus* nymphs, based on cross validation results from the final boosted regression tree models to predict tick abundance in southern Scandinavia. The red line is the actual regression line from a linear regression analysis run on the observed and predicted values, whereas the black line depicts a 1:1 relationship between observed and predicted values. NRMSE is the normalized root-mean-square error.

The best BRT tuning parameters for all instar models are found in Table 3. CV for the final larva model produced a NRMSE of 0.69 and a R^2^ of 0.52 (Fig. 3a), whereas the final model including all data produced a NRMSE of 0.15 and a R^2^ of 0.98. CV for the final nymph model produced a NRMSE of 0.65 and a R^2^ of 0.58 (Fig. 3b), and the final model including all data produced a NRMSE of 0.21 and a R^2^ of 0.96. Both the final adult male and adult female model performed poorly due to low abundance counts in all 3 countries (Fig. 2, CV results males: NRMSE = 0.94, R^2^ = 0.10, CV results females: NRMSE = 0.96, R^2^ = 0.04). Even when pooled into one single adult group, the model performed poorly (CV results: NRMSE = 0.93, R^2^ = 0.11) and thus adult ticks were omitted from further prediction, IDW interpolation and mapping.

**Table 3:** The best BRT tuning parameters for all final instar models, validated using 5-fold cross validation with 10 repeats.

Instar model	Interaction depth	Learning rate	Minimum number of observations per node
Larvae	3	0.01	2
Nymphs	2	0.01	2
Adult males	2	0.001	2
Adult females	1	0.001	2
Adults	2	0.001	2

**Figure 4:**
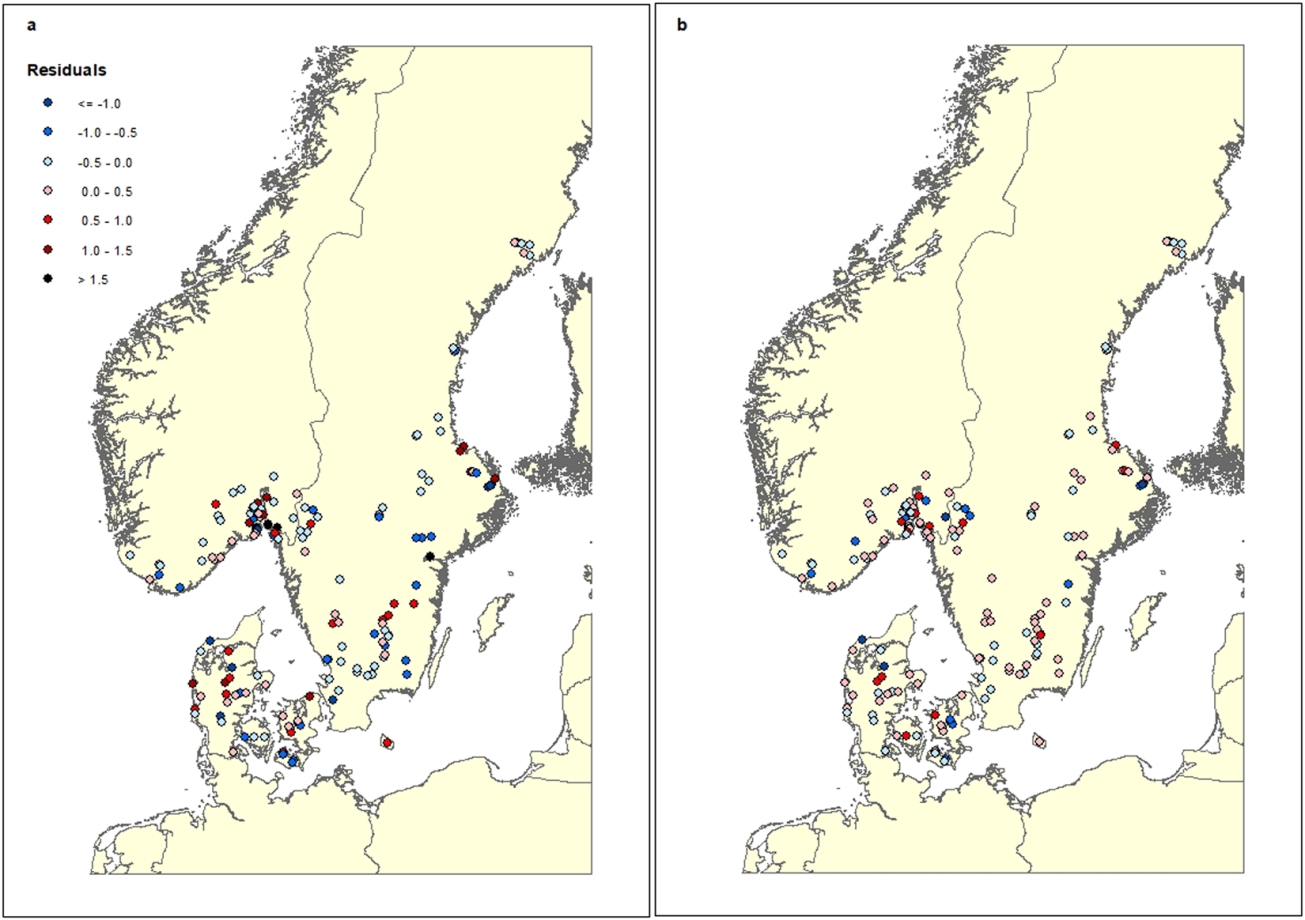
Prediction errors for *I. ricinus* larvae and nymphs. The map depict the predicted values (observed abundance, log(N + 1)) – predicted abundance (log(N + 1)) of (**a**) *I. ricinus* larva abundance and (**b**) *I. ricinus* nymph abundance, based on the final boosted regression tree models to predict abundance in southern Scandinavia. High negative values show sites where predicted abundance is much higher than the measured abundance of nymphs or larvae, whereas high positive values indicate sites with low predicted abundance, but with measured higher abundance of tick larvae or nymphs.

**Figure 5:**
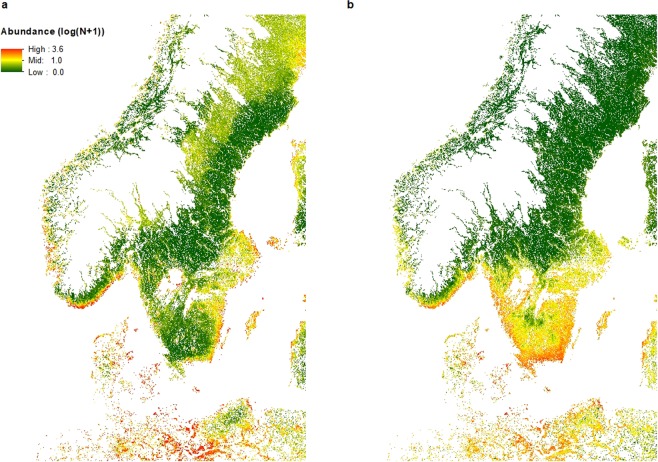
Predicted abundance maps for *I. ricinus* larvae and nymphs. Maps of predicted abundance (log(N + 1)) for (**a**) *I. ricinus* larvae and (**b**) *I. ricinus* nymphs, produced by the final boosted regression tree models. The mapped region encompasses 100%, 68.4% and 85.8% of Denmark’s, Norway’s and Sweden’s total land area. White areas within Denmark, Norway and Sweden are altitudes above 450 m or lakes, rivers and streams, or habitats other than our sampled forest and meadow habitats (not predicted).

We plotted the prediction errors for both the larva and nymph model (observed log(abundance + 1) minus mean predicted log(abundance + 1)) over the folds and the repeats, in order to visualize a potential spatial pattern in the prediction errors and concluded there were no specific patterns (Fig. 4). The final prediction maps encompassed 100%, 68.4% and 85.8% of Denmark, Norway and Sweden’s total land area (Fig. 5). Larva and nymph abundance predictions for Denmark were generally higher than for Norway and Sweden, with maximum predicted abundance being almost 3.5–4 times higher than for Norway and Sweden (Table 4). The average abundance for larvae was approximately double of the nymph abundance for all three countries (Table 4).

**Table 4:** Summary data of the predicted abundances within the study region for each of Denmark, Norway and Sweden.

Country	Instar	Min	Max	Mean	Stdev
Denmark	Larvae Nymphs	0 0	4,265 250	30 14	4 2
Norway	Larvae Nymphs	0 0	1,121 111	2 1	2 2
Sweden	Larvae Nymphs	0 0	1,258 161	2 1	2 3

Abundances are back-transformed from log(N + 1) values and are abundances/400 m^2^ (corresponding to the area traversed using the transects, see Methods). All values have been rounded to whole numbers. Min = minimum number predicted, Max = maximum number predicted, Mean = mean number predicted, Stdev = standard deviation of the mean number predicted. Minimums are corrected from −1 to 0, values below zero arising from using gaussian regression in the BRT models.

**Figure 6:**
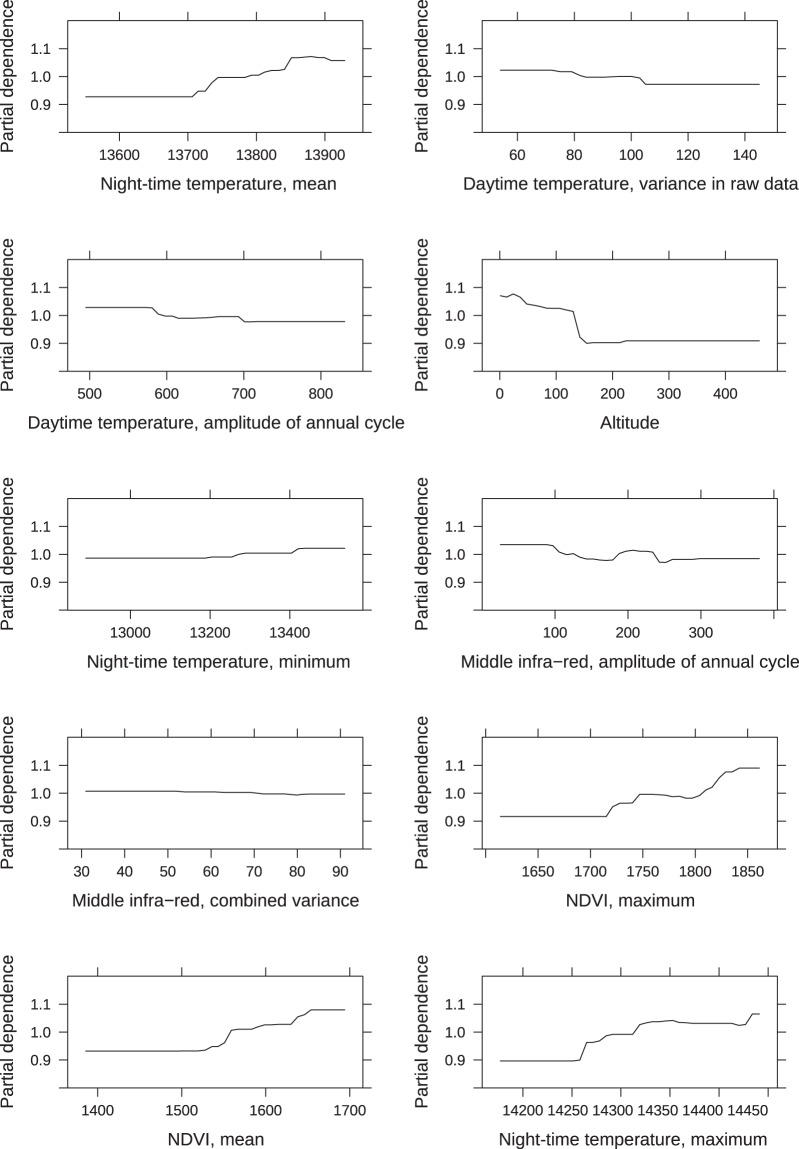
Predictor importance for the *I. ricinus* larva model. Plots of the 10 most important predictors in the final boosted regression tree model predicting larval *I. ricinus* distribution in southern Scandinavia. All are partial dependence plots, and illustrates the marginal effect of the selected variables on the response after integrating out the other variables. All daytime and night-time temperatures are land surface temperatures. NDVI is the normalized difference vegetation index.

**Figure 7:**
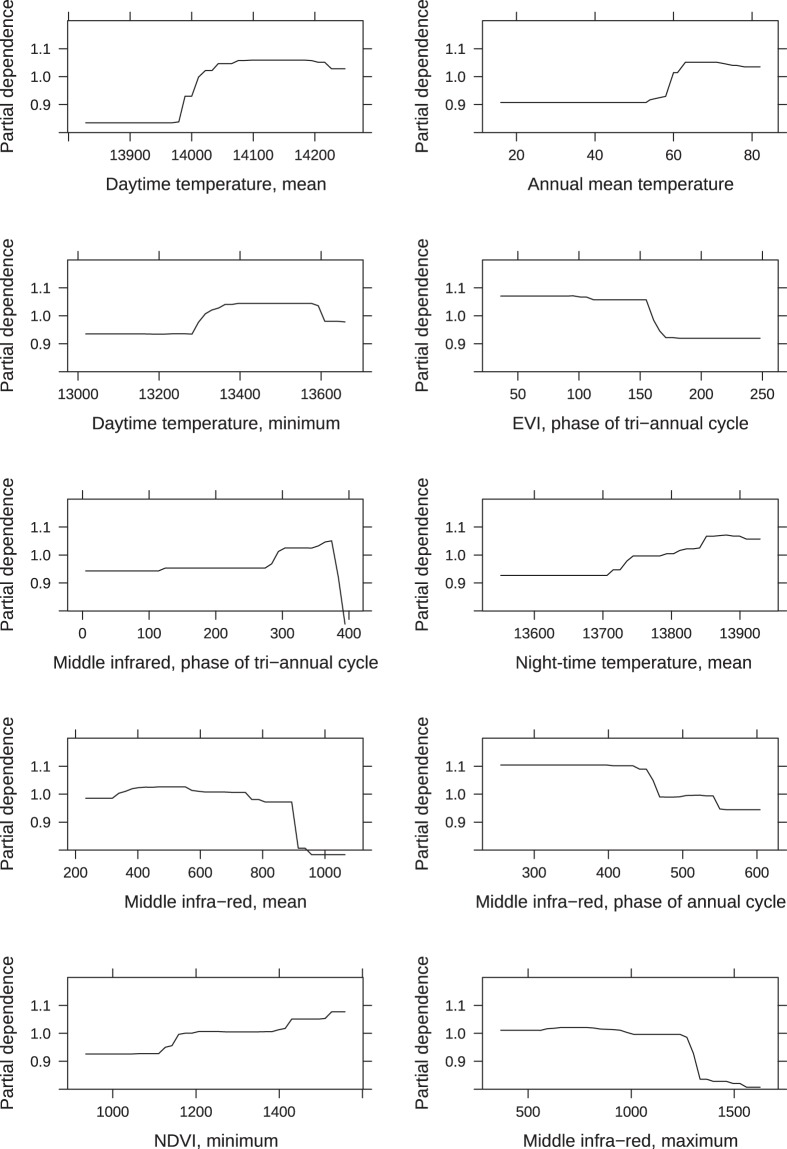
Predictor importance for the *I. ricinus* nymph model. Plots of the 10 most important predictors in the final boosted regression tree model predicting nymphal *I. ricinus* distribution in southern Scandinavia. All are partial dependence plots, and illustrates the marginal effect of the selected variables on the response after integrating out the other variables. All daytime and night-time temperatures are land surface temperatures. NDVI is the normalized difference vegetation index and EVI is the enhanced vegetation index.

For both the nymph and larva models, the most important predictors were day and night-time land surface temperatures and related parameters, the middle infra-red index and related parameters, and parameters related to the vegetation indexes EVI and NDVI (Figs. 6, 7). In the larva model, altitude was the fourth most important predictor (Fig. 6), whereas it ranked 11 in the nymph model. For both the larva and nymph model, land cover, habitat fragmentation and edge length ranked fairly low in importance; > rank 13 for the larva model and > rank 14 for the nymph model.

**Figure 8:**
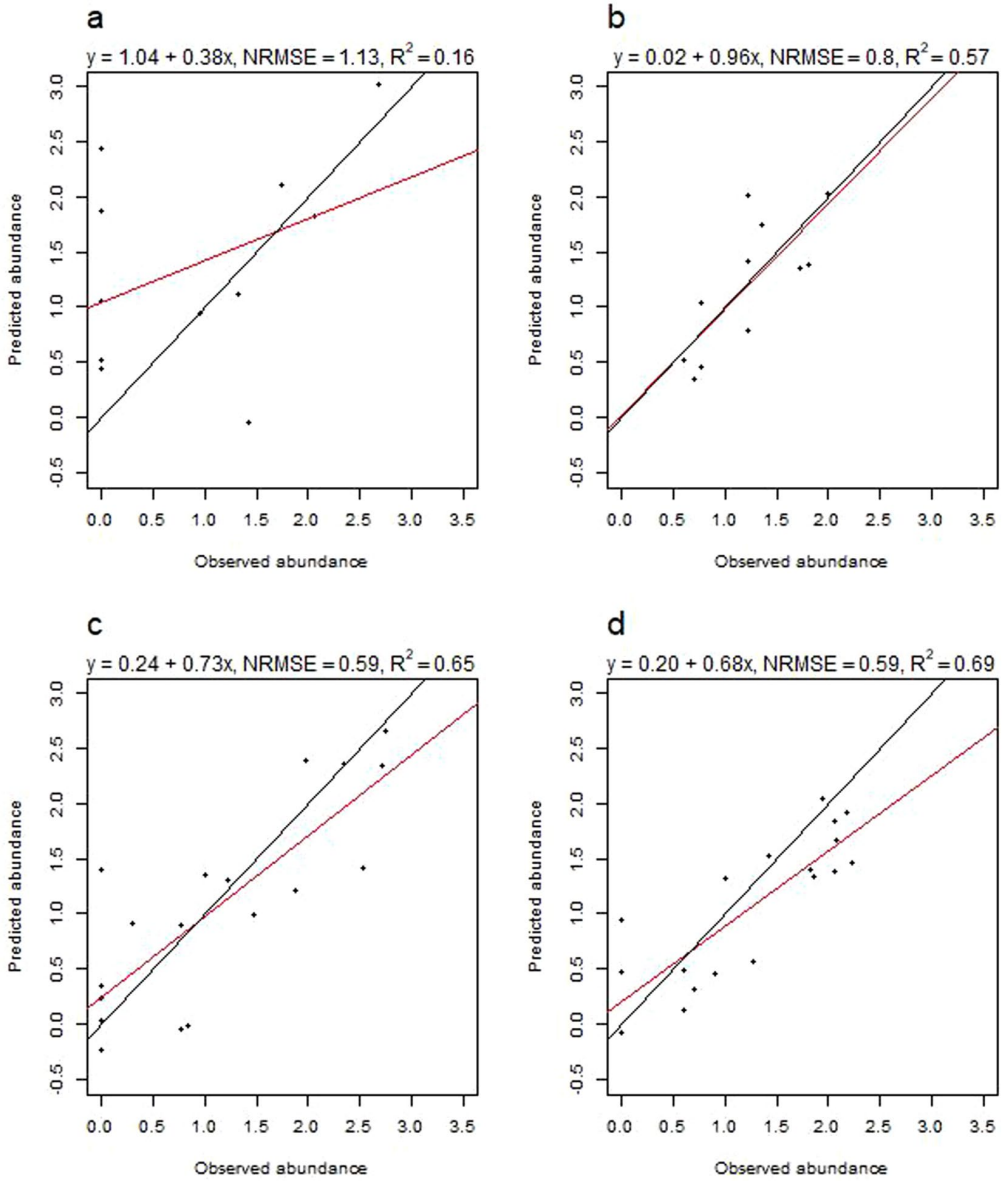
Observed versus predicted abundances of *I. ricinus* larvae and nymphs for 2017 sites. Observed abundances (log(N + 1)) plotted against predicted abundances for (**a**) *I. ricinus* larvae at new 2017 sites, (**b**) *I. ricinus* nymphs at new 2017 sites, (**c**) *I. ricinus* larvae at revisited 2017 sites, and (**d**) *I. ricinus* nymphs at revisited 2017 sites. Predictions were calculated using boosted regression tree models trained with 2016 data to predict tick abundance in southern Scandinavia. The red line is the actual regression line from a linear regression analysis run on the observed and predicted values, whereas the black line depicts a 1:1 relationship between observed and predicted values. NRMSE is the normalized root-mean-square error.

Testing the models on data from the 12 new sites in 2017, resulted in NRMSE = 1.13 and R^2^ = 0.16, and NRMSE = 0.80, R^2^ = 0.57 for the larva and nymph model respectively (Fig. 8a,b). The 18 larva models used to test each revisited 2017 site gave 5-fold CV results with NRMSE values ranging from 0.68 to 0.71, with corresponding R^2^ values ranging from 0.50 to 0.54. For the nymph models, the NRMSE values ranged from 0.65 to 0.68, with corresponding R^2^ values ranging from 0.54 to 0.57. Testing model fit when predicting the 2017 revisited sites, produced NRMSE = 0.59 and R^2^ = 0.65 for the larva model and NRMSE = 0.59 and R^2^ = 0.69 for the nymph model (Fig. 8c,d).

### Simple spatial interpolation

**Figure 9:**
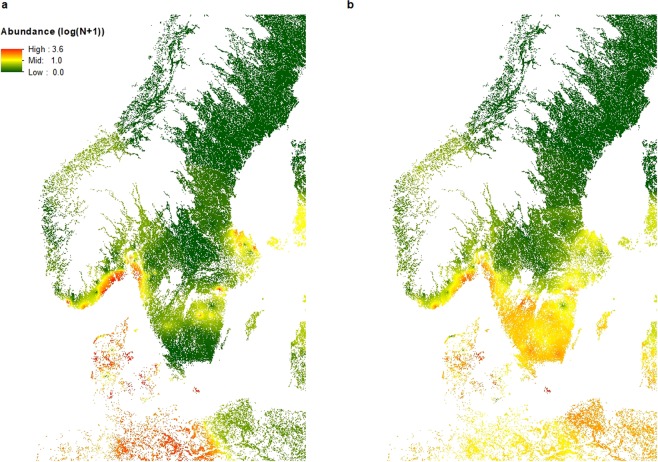
Interpolation maps of *I. ricinus* larvae and nymphs. Maps of interpolated abundance (log(N + 1)) for (**a**) *I. ricinus* larvae and (**b**) *I. ricinus* nymphs, produced by Inverse Distance Weighted interpolation. The mapped region encompasses 100%, 68.4% and 85.8% of Denmark’s, Norway’s and Sweden’s total land area. White areas within Denmark, Norway and Sweden are altitudes above 450 m or lakes, rivers and streams, or habitats other than our sampled forest and meadow habitats (not interpolated).

The best tuning parameters for the larval IDW model was a power of 1.2 and a neighborhood search of 10 points. CV results produced a NRMSE of 0.82 and a R^2^ of 0.34. For the nymph model, the best tuning parameters was a power of 1 and a neighborhood search of 8 points. CV results produced a NRMSE of 0.81 and a R^2^ of 0.34. As with the BRT prediction maps, we only predicted to habitat types that corresponded to our sampling sites (forest and meadow, Table 1) and only altitudes below 450 m^34^ (Fig. 9).

## Discussion

We developed models to predict the abundance of *I. ricinus* nymphs and larvae in southern Scandinavia using ML techniques and environmental variables, and created abundance maps of the region for the period August-September, 2016. Both models performed well and outperformed simple spatial interpolation methods; however the nymph model performed slightly better than the larva model with CV and the nymph model was also better at predicting abundance the following year.

Previously, we have modeled the spatial distribution of nymphs in southern Scandinavia using models with high predictive power^34^. We found annual variation in tick abundance between 2016 and 2017, but the BRT abundance models still had high predictive power for the revisited sites in 2017. The results showed that the temporal variation between years was small enough to give our models high predictive power for both 2016 and revisited sites the following year. The model predictions for the new sites in 2017 performed well for the nymph model, whereas the larva model performed poorly. We tried correcting the 2017 larva data, using a 2016:2017 ratio based on the geometric mean, but this did not improve the 2017 predictions. The poor predictive power of the larva model for the new sites in 2017 may partly be attributed to a high ratio of larval absences at the new sites in 5 out of 12 sites, making this dataset zero-inflated, and thus not suitable for the Gaussian regression used in the BRT model. We found large spatial differences in the abundances of larvae in both years. As reported from other field studies^49,50^, we found larvae to be more patchily distributed than nymphs, a pattern that might be explained by the *I. ricinus* life cycle. The presence and abundance of larvae depends on where tick eggs are deposited. Larvae are not highly mobile^3^, and as one female *I. ricinus* can lay around 2000 eggs^51,52^, all larvae counted in one patch could potentially stem from a single female. Nymphs, however, are found where a larva has dropped of a host and has metamorphosed into a nymph and thus the distribution of nymphs depends more on host distribution and movement^3^. One caveat with using our 2016 models to predict abundance to the new sites in 2017, was that the new sites were not randomly selected, but based on areas of interest. This could potentially have resulted in biases in the evaluation of our models for the 12 new 2017 data, but not for the 18 revisited sites.

The BRT models had a tendency to overestimate abundance at sites with zero abundance (Fig. 3). The repeated CV predictions in a BRT model is the average taken from many models, thus the models tend to overestimate low values and underestimate high values^53^. To overcome this problem, we explored splitting the data into a presence/absence binomial (Bernoulli) BRT model and a log-transformed presence only Gaussian BRT^53,54^, however the resulting ensemble models performed much poorer than the one-model approach and were therefore abandoned.

**Figure 10:**
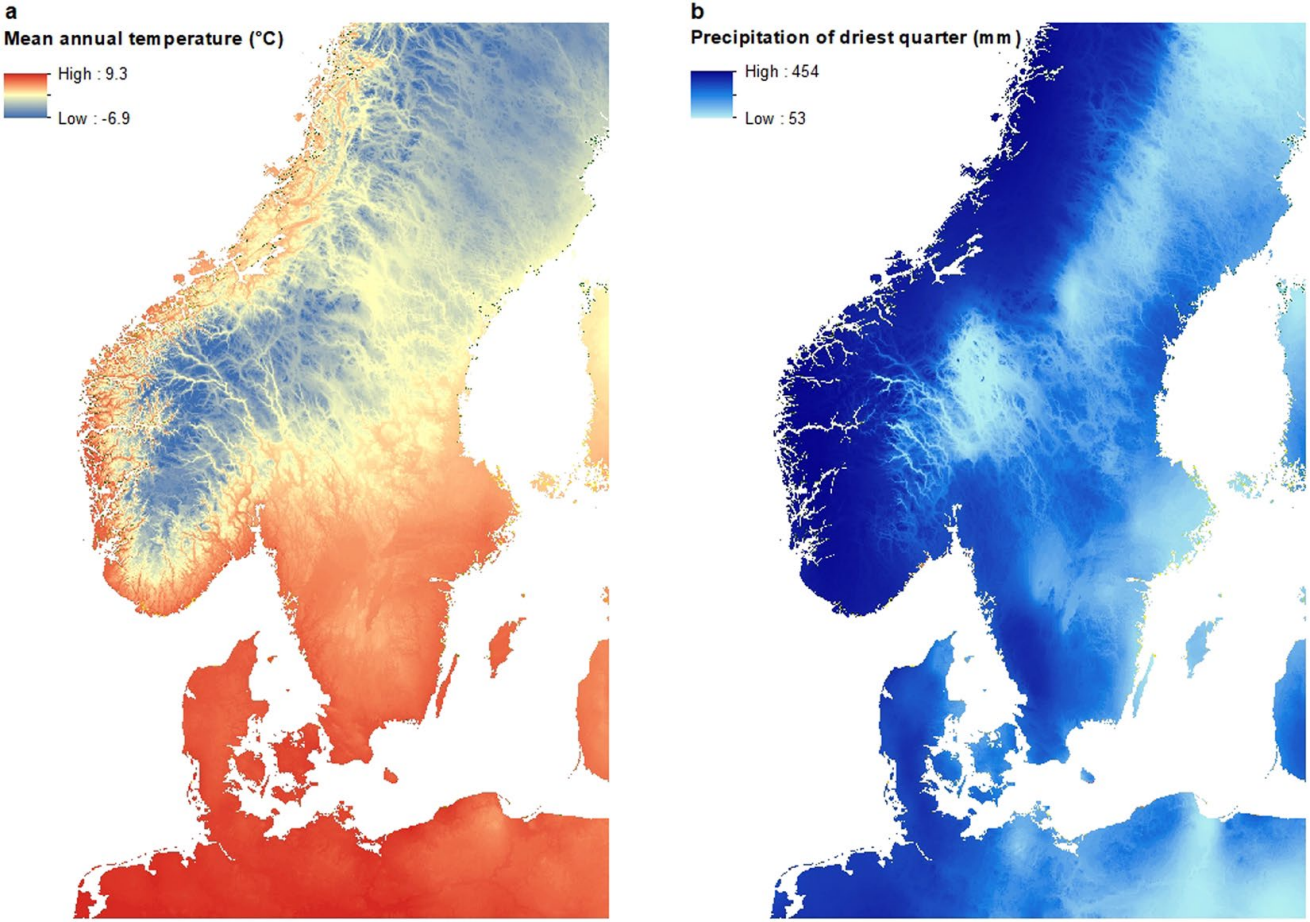
Temperature and precipitation maps. (**a**) Map of mean annual temperature in Scandinavia and (**b**) Map of precipitation of driest quarter (BIO1 and BIO17 from BioClim, see Table 2 in the manuscript).

The nymph prediction map, based on the 2016 field data, corresponded well with the known distribution of ticks in Scandinavia, with high abundance throughout Denmark (average prediction value ca. 11–17 times higher than in either Norway or Sweden), along the southern coast of Norway and southern parts of Sweden^3,6,7,11,55,56^. As in Kjær *et al*.^34^, we noticed a distinct border above the great lakes in Sweden, where abundance was lower, coinciding with the biogeographical and climatic boundary called Limes Norrlandicus (LN)^57^. Above the LN we find the Boreal zone, whereas below it we find the more species rich boreo-nemoral zone with shorter and milder winters^57^. The prediction map is mainly temperature driven and the higher abundances in the south of Sweden and around the coastline of Norway distinctly follows a temperature gradient, which may also explain the distinct patches of low predicted abundance below the great lakes in Sweden, where we observe lower mean annual temperatures (Fig. 10a). In Denmark we predicted high nymph abundance throughout the country except in the dry heathlands and sandy habitats of central and western Jutland, which corresponds to what we know about tick biology and the need for a high relative humidity and soil moisture to sustain ticks^3^.

The larva prediction map showed a slightly different pattern for Sweden, particularly in south-central Sweden, where larval abundance was low compared to relatively high abundances of nymphs. This pattern was also found in the observed abundances, and could potentially be explained by the area being located 200–250 meters above sea level (MASL) with its highest point being 377 MASL. The highland climate is generally colder with more snow and consequently the vegetation and fauna differs from the rest of the surrounding lower areas of southern Sweden, possibly producing spurious effects on larva survival- and life stage turn-over rates. We did find altitude to be among the top 5 predictors for the larval model. Previous studies from the Czech Republic have shown that *I. ricinus* egg survival and hatching decreases with altitude^58^, and even though we only surveyed sites with altitudes below 450 m, the model predicted lower larval abundance at higher altitudes (Fig. 6). The higher larva abundance in the northern parts of Sweden could potentially be due to the precipitation in the driest quarter variable (ranked 17 in variable importance for the larval model, Fig. 10b), with the higher abundance band corresponding to a band with lower precipitation.

Temperature was the main driver of both the nymph and larva abundance model. However, the BRT modelling method cannot detect causality and thus we cannot tell if this effect of temperature is direct or due to other factors correlated with temperature, such as host species distribution and abundance^3,7,29^. None the less, we were able to create biologically feasible prediction maps based on environmental predictors alone. Contrary to what we know about the effect of land cover, landscape fragmentation and forest edge on tick densities, the landscape cover and configuration variables did not rank high in importance for either model. It may be that the 100 m^2^ and 1 km^2^ resolutions we used were not fine-scaled enough to catch the true landscape configuration. We did see an effect of NDVI and EVI on both larva and nymph abundance (Figs. 6, 7), which also reflects the amount and type of land cover as these indicators are both based on visible and near-infrared sunlight reflected by plants.

The IDW interpolation maps relied solely on the abundance values of the surrounding locations assuming that high and low abundance were spatially correlated. Compared to the BRT maps, the IDW maps failed to predict the higher larval abundance around the Norwegian and Swedish coastlines, and did not predict a lower nymph abundance below the great Swedish lakes or the higher abundance on the west coast of Norway. The IDW methods had lower predictive power than our BRT models, indicating that climatic and environmental predictors may be among the key drivers of *I. ricinus* abundance.

With temperature as one of the key drivers of tick abundance and distribution, changes in temperature may not only directly affect tick and host survival^7^ but may also influence plant communities and the timing of the growing season. Studies in southern Sweden have shown that increases in tick abundance from the 1980s to the 1990s were related to extended spring and autumn seasons^59^, and scenario models for Sweden found that future climate change may cause a markedly prolonged vegetation period causing an increase in both range and abundance of *I. ricinus*^29^. In Scandinavia, a range expansion alone may however not markedly increase human exposure to ticks. In Kjær *et al*.^34^, we related tick distribution to human population density data in Scandinavia and found that a potential *I. ricinus* range expansion may not affect many additional people – as ca. 70–80% of the Scandinavian population already live within the distribution range of *I. ricinus*. However, an increase in abundance within the already established range is likely to have an effect on human exposure to tick-borne pathogens, as risk of pathogen transmission is related to the actual abundance of ticks, densities of reservoir hosts and the pathogen rates within these reservoirs^1,16,17,20,21^. Even though changes in temperature and climate may cause changes to tick abundance, this may not necessarily create an increase in pathogen prevalence as pathogen transmission also depends on the tick life cycle^60^. TBEV is thought to be transmitted through co-feeding on the same host^1^, and this has been suggested to require the presence of different stages of ticks (larvae and nymphs) on the same host, and so is dependent on synchrony in the seasonal activity of these instars^1,35^. Climate change may disrupt the synchrony of seasonal activity of larvae and nymphs and thus disrupt the transmission through co-feeding^1,35^. To make more accurate predictions for the future, there is a great need to better understand the different pathogen transmission pathways in tick vectors, how they contribute to overall pathogen prevalence, and how these pathways may be affected by climate.

Using environmental variables, we created models and maps of tick nymph and larva abundance with relatively high performance for the year, the tick data were collected. The data used to create the models give us abundance for a moment in time, and as such do not capture the annual variation in tick abundance or the effect of climate change on future abundance. We were, however, able to accurately predict to revisited sites in 2017 for both larvae and nymphs, and for entirely new sites in 2017, the nymph model was able to predict fairly accurately. Our results indicate that temperature affects the abundance of *I. ricinus* ticks, and thus a change in temperature in the coming decades may affect the abundance and distribution of *I. ricinus* in Scandinavia and other areas. A change in tick abundance may affect the transmission of pathogens and alter the existing human exposure risk and tick-borne disease incidence. Data on tick abundance, as produced in our models, may be used in epidemiological models and may aid authorities in determining areas to be targeted for surveillance and guide citizens and pet owners on where to take preventive measures. The modelling concept shown here, is a first and promising step towards more detailed models using dynamic weather data to predict tick abundance in the future.

## Data Availability

The datasets analysed during the current study are available from the corresponding author on reasonable request.

## References

[1] Estrada-Peña A, De J, de la Fuente J (2014). The ecology of ticks and epidemiology of tick-borne viral diseases. Antiviral Res..

[2] Jensen PM, Skarphédinsson S, Jensen PM, Kristiansen K (2005). Survey of tickborne infections in Denmark. Emerg. Infect. Dis..

[3] Medlock JM (2013). Driving forces for changes in geographical distribution of *Ixodes ricinus* ticks in Europe. Parasit. Vectors.

[4] Gage KL, Burkot TR, Eisen RJ, Hayes EB (2008). Climate and vectorborne diseases. Am. J. Prev. Med..

[5] Martens W, Jetten T, Rotmans J, Niessen L (1995). Climate change and vector-borne diseases: A global modelling perspective. *Glob. Environ*. Chang..

[6] Jore S (2011). Multi-source analysis reveals latitudinal and altitudinal shifts in range of *Ixodes ricinus* at its northern distribution limit. Parasit. Vectors.

[7] Jaenson TGT, Jaenson DGE, Eisen L, Petersson E, Lindgren E (2012). Changes in the geographical distribution and abundance of the tick *Ixodes ricinus* during the past 30 years in Sweden. Parasit. Vectors.

[8] Soleng A (2018). Distribution of *Ixodes ricinus* ticks and prevalence of tick-borne encephalitis virus among questing ticks in the Arctic Circle region of northern Norway. Ticks Tick. Borne. Dis..

[9] Andreassen A (2012). Prevalence of tick borne encephalitis virus in tick nymphs in relation to climatic factors on the southern coast of Norway. Parasit. Vectors.

[10] Lindgren E, Gustafson R (2001). Tick-borne encephalitis in Sweden and climate change. Lancet (London, England).

[11] Kjelland V (2018). Tick-borne encephalitis virus, *Borrelia burgdorferi* sensu lato, *Borrelia miyamotoi*, Anaplasma phagocytophilum and *Candidatus* Neoehrlichia mikurensis in *Ixodes ricinus* ticks collected from recreational islands in southern Norway. Ticks Tick. Borne. Dis..

[12] Paulsen KM (2015). Prevalence of tick-borne encephalitis virus in *Ixodes ricinus* ticks from three islands in north-western Norway. APMIS.

[13] Fomsgaard A, Christiansen CB, Bødker R (2009). First identification of tick-borne encephalitis in Denmark outside of Bornholm, August 2009. Eurosurveillance.

[14] Fomsgaard A (2013). Tick-borne encephalitis virus, Zealand, Denmark, 2011. Emerg. Infect. Dis..

[15] Del Fabbro S, Gollino S, Zuliani M, Nazzi F (2015). Investigating the relationship between environmental factors and tick abundance in a small, highly heterogeneous region. J. Vector Ecol..

[16] Jaenson TGT (2009). Risk indicators for the tick *Ixodes ricinus* and *Borrelia burgdorferi* sensu lato in Sweden. Med. Vet. Entomol..

[17] Hudson PJ (2001). Tick-borne encephalitis virus in northern Italy: molecular analysis, relationships with density and seasonal dynamics of *Ixodes ricinus*. Med. Vet. Entomol..

[18] Nazzi F (2010). Ticks and Lyme borreliosis in an alpine area in northeast Italy. Med. Vet. Entomol..

[19] Hubalek Z, Halouzka J, Juricova Z (2003). Longitudinal surveillance of the tick *Ixodes ricinus* for borreliae. Med. Vet. Entomol..

[20] Mysterud A (2018). Tick abundance, pathogen prevalence, and disease incidence in two contrasting regions at the northern distribution range of Europe. Parasit. Vectors.

[21] Jensen PM, Hansen H, Frandsen F (2000). Spatial Risk Assessment for Lyme Borreliosis in Denmark. Scand. J. Infect. Dis..

[22] Hvidsten D (2014). *Ixodes ricinus* and *Borrelia* prevalence at the Arctic Circle in Norway. Ticks Tick. Borne. Dis..

[23] Jensen PM, Jespersen JB (2005). Five decades of tick-man interaction in Denmark - An analysis. Exp. Appl. Acarol..

[24] Lindström A, Jaenson TGT (2003). Distribution of the Common Tick, *Ixodes ricinus* (Acari: Ixodidae), in Different Vegetation Types in Southern Sweden. J. Med. Entomol..

[25] Horobik V, Keesing F, Ostfeld RS (2006). Abundance and *Borrelia burgdorferi*-infection prevalence of nymphal *Ixodes scapularis* ticks along forest-field edges. Ecohealth.

[26] Tack W (2012). Local habitat and landscape affect *Ixodes ricinus* tick abundances in forests on poor, sandy soils. For. Ecol. Manage..

[27] Mejlon HA, Jaenson TGT (2009). Jaenson (1993) Seasonal Prevalence of *Borrelia burgdorferi* in *Ixodes ricinus* in Different Vegetation Types in Sweden. Scand. J. Infect. Dis..

[28] Walhström LK, Kjellander P (1995). Ideal free distribution and natal dispersal in female roe deer. Oecologia.

[29] Jaenson TGT, Lindgren E (2011). The range of *Ixodes ricinus* and the risk of contracting Lyme borreliosis will increase northwards when the vegetation period becomes longer. Ticks Tick. Borne. Dis..

[30] Jore S (2014). Climate and environmental change drives *Ixodes ricinus* geographical expansion at the northern range margin. Parasit. Vectors.

[31] Estrada-Peña A (2013). Association of environmental traits with the geographic ranges of ticks (Acari: Ixodidae) of medical and veterinary importance in the western Palearctic. A digital data set. Exp. Appl. Acarol..

[32] Brownstein JS, Holford TR, Fish D (2003). A climate-based model predicts the spatial distribution of the Lyme disease vector *Ixodes scapularis* in the United States. Environ. Health Perspect..

[33] Estrada-Peña A (1998). Geostatistics and Remote Sensing as Predictive Tools of Tick Distribution: a Cokriging System to Estimate *Ixodes scapularis* (Acari: Ixodidae) Habitat Suitability in the United States and Canada from Advanced Very High Resolution Radiometer Satellite Imagery. J. Med. Entomol..

[34] Kjær LJ (2019). Predicting and mapping human risk of exposure to *Ixodes ricinus* nymphs using climatic and environmental data, Denmark, Norway and Sweden, 2016. Eurosurveillance.

[35] Randolph SE, Rogers DJ (2000). Fragile transmission cycles of tick-borne encephalitis virus may be disrupted by predicted climate change. Proc. Biol. Sci..

[36] Zeimes CB, Olsson GE, Hjertqvist M, Vanwambeke SO (2014). Shaping zoonosis risk: landscape ecology vs. landscape attractiveness for people, the case of tick-borne encephalitis in Sweden. Parasit. Vectors.

[37] Porretta D (2013). Effects of global changes on the climatic niche of the tick *Ixodes ricinus* inferred by species distribution modelling. Parasit. Vectors.

[38] Alkishe AA, Peterson AT, Samy AM (2017). Climate change influences on the potential geographic distribution of the disease vector tick *Ixodes ricinus*. PLoS One.

[39] Furlanello, C. *et al*. GIS and the Random Forest Predictor: Integration in R for Tickborne Disease Risk Assessment. in *Proceedings of the 3rd International Workshop on Distributed Statistical Computing (DSC 2003)* (eds. Kurt, H., Leisc, F. & Zeileis, A.) 1–11 (2014).

[40] Scharlemann JPW (2008). Global Data for Ecology and Epidemiology: A Novel Algorithm for Temporal Fourier Processing MODIS Data. PLoS One.

[41] Corine Land Cover 2006 raster data [Internet]. Available at, https://www.eea.europa.eu/data-and-maps/data/clc-2006-raster (2010).

[42] R Development Core Team. R: A Language and Environment for Statistical Computing (2017)

[43] Gray JS, Lohan G (1982). The development of a sampling method for the tick *Ixodes ricinus* and its use in a redwater fever area. Ann. Appl. Biol..

[44] Elith J, Leathwick JR, Hastie T (2008). A working guide to boosted regression trees. J. Anim. Ecol..

[45] ESRI. ArcGIS Desktop: Release 10.6.1 Redlands, CA: Environmental Systems Research Institute (2017).

[46] Shepard, D. D. A two-dimensional interpolation function for irregularly-spaced data. In *Proceedings of the 1968 23rd ACM National Conference* 517–524, 10.1145/800186.810616 (ACM Press, 1968).

[47] Tälleklint-Eisen L, Lane RS (2000). Spatial and Temporal Variation in the Density of *Ixodes pacificus* (Acari: Ixodidae) Nymphs. Environ. Entomol..

[48] Daniel M, Malý M, Danielová V, Kříž B, Nuttall P (2015). Abiotic predictors and annual seasonal dynamics of *Ixodes ricinus*, the major disease vector of Central Europe. Parasit. Vectors.

[49] Paul REL (2016). Environmental factors influencing tick densities over seven years in a French suburban forest. Parasit. Vectors.

[50] Tack W, Madder M, Baeten L, De Frenne P, Verheyen K (2012). The abundance of *Ixodes ricinus* ticks depends on tree species composition and shrub cover. Parasitology.

[51] Gray JS (1981). The Fecundity of *Ixodes Ricinus* (L.) (Acarina: Ixodidae) and the Mortality of its Developmental Stages Under Field Conditions. Bull. Entomol. Res..

[52] Dobson ADM, Finnie TJR, Randolph SE (2011). A modified matrix model to describe the seasonal population ecology of the European tick *Ixodes ricinus*. J. Appl. Ecol..

[53] Smith, A. N. H., Duffy, C. A. J. & Leathwick, J. R. Predicting the distribution and relative abundance of fishes on shallow subtidal reefs around New Zealand.

[54] Froeschke JT, Froeschke BF (2016). Two-stage boosted regression tree model to characterize southern flounder distribution in Texas estuaries at varying population sizes. Mar. Coast. Fish..

[55] Mehl R (1983). The distribution and host relations of Norwegian ticks (Acari, Ixodides). Fauna Nor. Ser. B.

[56] Larsen AL (2014). Detection of specific IgG antibodies in blood donors and tick-borne encephalitis virus in ticks within a non-endemic area in southeast Norway. Scand. J. Infect. Dis..

[57] Sjörs, H. *Nordisk växtgeografi (In Swedish)*. (Bonniers, Scandinavian university books, 1967).

[58] Materna J, Daniel M, Metelka L, Harčarik J (2008). The vertical distribution, density and the development of the tick *Ixodes ricinus* in mountain areas influenced by climate changes (The Krkonoše Mts., Czech Republic). Int. J. Med. Microbiol..

[59] Lindgren E, Tälleklint L, Polfeldt T (2000). Impact of Climatic Change on the Northern Latitude Limit and Population Density of the Disease-Transmitting European Tick *Ixodes ricinus*. Environ. Health Perspect..

[60] Ogden NH, Lindsay LR (2016). Effects of Climate and Climate Change on Vectors and Vector-Borne Diseases: Ticks Are Different. Trends Parasitol..

[61] WorldClim 1.4 1960-1990 raster data [internet]. Available at, http://www.worldclim.org/current (2005).

[62] Bioclimatic variables | WorldClim - Global Climate Data 1960-1990 [Internet]. Available at, http://www.worldclim.org/bioclim. (Accessed: 18th February 2019).

[63] Harmonized world soil database v1.2 [Internet]. 2009. Available at, http://www.fao.org/soils-portal/soil-survey/soil-maps-and-databases/harmonized-world-soil-database-v12/en/. (Accessed: 18th February 2019).

